# The Influence of HEMC on Cement and Cement-Lime Composites Setting Processes

**DOI:** 10.3390/ma13245814

**Published:** 2020-12-20

**Authors:** Edyta Spychał, Przemysław Czapik

**Affiliations:** Faculty of Civil Engineering and Architecture, Kielce University of Technology, 25-314 Kielce, Poland; p.czapik@tu.kielce.pl

**Keywords:** cement, hydrated lime, HEMC, mortar, paste, hydration, setting process, ultrasonic technique

## Abstract

In this article, the effect of hydroxyethyl methyl cellulose (HEMC), which is a polymeric viscosity modifying admixture on the mineral based composites setting processes, was studied. Previous studies available in the literature included the evaluation of the influence of this admixture on the hydration processes of cement or lime pastes. In this paper, the analysis of this issue was extended to include cement-lime composites. The composition of the pastes and mortars differed in the type of binder (the tests were carried out on cement-based and cement-lime-based materials, in which the cement was replaced in 50% with hydrated lime), as well as the amount and viscosity of the admixture. The study of mortars setting processes and hardening processes using the ultrasonic method was supplemented in the work with calorimetric measurements and phases analysis by the X-ray diffraction method. Finally, it was found that the HEMC reduces the rate of a hydration reaction in cement and cement-lime pastes. The amount of admixture used has a greater influence on the changes taking place during the setting process than the admixture viscosity or the type of binder.

## 1. Introduction

The use of chemical admixtures in modern mortar technology is a prerequisite for obtaining materials of high quality and durability that meet the standard requirement. The aim is to ensure that the mortars are characterized by good workability, ease of application, adequate adhesion to the masonry, and lowest possible shrinkage deformation. 

Cellulose ethers (CE) are a group of polymer admixtures used in dry pre-mix mineral-based binder materials [1,2,3]. Hydroxyethyl methyl cellulose (HEMC) in addition to hydroxypropyl methyl cellulose (HPMC) is one of the most popular cellulose ethers [3,4,5]. They are a group of admixtures, with the use of which it is possible to modify the viscosity of pastes and mortars, and, thus, change their rheological or application properties [1,2,5,6,7,8,9,10,11,12,13,14], which is important to know how this admixture influences the properties of fresh mortars. The research [2,3,7,8,14,15] showed that the cellulose ethers have an impact on the increase of water retention. Bülichen et al. [3] found that, at low dosages, HEMC achieves water retention by intramolecular sorption of water and concomitant swelling while, at a higher amount of admixture, the molecules agglomerate into large, hydrocolloidal microgel particles. This admixture changes the material rheological properties including plastic viscosity and yield stress [1,3,5,6,12,14]. The high-water retention capacity of the mortar, which can be obtained by using these polymers, prevents rapid water loss from the material and the drainage of some water from the mortar to the substrate. Thanks to this, it is possible to obtain greater adhesion and reduce shrinkage deformations [16,17]. On the other hand, cellulose ethers can cause a delay of hydration processes, as a result of which the setting and hardening time of the binders and mortars become longer [1,18,19,20,21,22,23,24,25,26,27]. According to Hua et al. [25], the retardation effect of cellulose ethers favors the increase of operating time and the decrease of the consistency or slump loss of freshly mortars, but possibly leads to unexpected delay during construction. Study of the influence of this admixture on cement hydration kinetics can facilitate when controlling cement hydration, which is important, especially for application. Izaguirre et al. [27] tested two different additives (cellulose ether and guar gum), which were added to lim-based mortars. Among other things, the setting time was tested. The obtained results showed an increase in setting time from 3 h 15 min for the reference mortar to 10 h 30 min for mortar with an admixture. An adsorption mechanism of this admixture on the Ca(OH)_2_ crystals was reported to reduce its entanglement between chains and, hence, the viscosity of the pastes as well as its water retention ability. Similar results of the influence on the delay of setting time were observed in ceramic tile adhesive mortars by Petit and Wirquin [1]. Initial setting time increased from 4 h for the reference mortar to 12 h 25 min for mortar modified with an admixture, but final setting time changed from 5 h 25 min to 18 h. Pourchez et al. [19] studied the influence of cellulose ethers on the hydration of C_3_A and C_3_A-sulphate systems. It was observed that this admixture leads to a gradual slowing down of the C_3_A hydration depending on cellulose ether chemistry. Two types of cellulose ethers were used in their research: hydroxypropyl methyl cellulose (HPMC) and hydroxyethyl cellulose (HEC). They observed that HECs include a higher delay than HPMCs. In their opinion, the substitutions group seems to be more important for controlling factors on C_3_A hydration rather than molecular mass. Betioli et al. [24] observed that cellulose ether HEMC did not change the typical profile of isothermal calorimetry curves. However, it decreased the rate of heat evolution during the acceleration period and decreased the maximum peak. It was expected that the induction period was importantly extended with a hydroxyethyl methyl cellulose (HEMC) admixture. The total heat output after 4 h of hydration was reduced (even 54% for paste with 0.5% HEMC). Oscillatory rheometry and isothermal calorimetry were used for showing the influence of cellulose ether on a hydration process. A steric dispersant barrier effect during the first two hours of hydration associated with a cement retarding nature was displayed, which resulted in reducing the setting speed. Authors of this article observed that the rheology of cement paste changes during the induction period and it was caused by the agglomeration of the particles. Pichniarczyk [22] concluded that methylcellulose has a significant influence on the early hydration of cement. However, that effect practically disappears after a longer period. The XRD (X-Ray Diffraction) pattern of cement paste with cellulose ether admixture peaks of portlandite and ettringite were of significantly lower intensity after 24 h of hydration, but gypsum peaks were of high intensity. These test results indicate a slower hydration process in pastes modified with methylcellulose. After 168 h, hydration differences in the intensity of portlandite and ettringite peaks were lower. X-ray diffraction was complemented with microstructure observations under the SEM. Knapen and Gemert [23] observed that the presence of water-soluble polymers in cement mortars influences the rate and degree of cement hydration as well as the nature and amount of hydration products. The heat evolution was measured by isothermal calorimetry measurements. It was found that the start of the acceleration stage of hydration was postponed by about 30 min in the MC (methyl cellulose) modified pastes (1% MC, HEC cement pastes) and by more than 5 h in the HEC modified paste. The induction period and the dormant period were extended. The rate of the following hydration reactions was slowed down. What was confirmed by lower values of the maximum heat release and broader exothermal peaks in the isothermal calorimetric curves of the cellulose ethers modified pastes.

The correct selection of the admixture, especially its quantity and viscosity, affects the parameters of the finished product. Knowledge of the processes taking place during the setting and hardening of mortars modified with this admixture allows for a correct and comprehensive assessment of its effectiveness and applicability. 

Based on the publications available in the literature, it can be concluded that the research results presented so far focus mainly on determining the influence of cellulose ethers on the rheological, physical-mechanical properties of cement [3,5,6,24] or lime [7,8,13,28] pastes and mortars. The cement hydration processes or its impact on the individual phases are also assessed. The subject of setting processes of mortars modified with a cellulose ether admixture focuses on cement-based materials. There is no explanation of the mechanism of action of this admixture and its influence on the hydration of pastes with a cement-lime binder. Therefore, this issue is the subject of these studies. Moreover, in publications for the evaluation of hydration processes, calorimetric measurements, XRD method, or conductometric measurements are usually used [21,22,24,28,29]. In this publication, the authors performed an analysis of the influence of the admixture on the setting processes of mortars, using the ultrasonic technique. Applying the proposed method of examinations, it is possible to describe the rate of setting and hardening of materials based on cement and cement-lime mortars with high accuracy due to the fact that measurements are made on an ongoing basis, which can make it possible to monitor and control the properties of mortars in the plastic state. Using the knowledge gained on the basis of an ultrasonic technique, it is possible to evaluate the influence of individual components on the parameters of the mortar or paste. The results of the ultrasonic technique complement the standard tests. In addition, they allow us to predict the mechanical properties of mortars [30].

## 2. Materials and Methods

### 2.1. Materials

In the studies, Portland cement CEM I 42.5 R (Cemex, Chełm, Poland), hydrated lime deeply separated (Alpol, ZSChiM "PIOTROWICE II", Sitkówka, Poland), two fractions of quartz sand 0.1–0.5 mm and 0.2–0.8 mm (Grudzeń Las, Grudzeń Las, Poland), and polymer admixture in the form of HEMC (WALOCEL, The Dow Chemical Company, Midland, MI, USA) were used. Cellulose ether used in tests is a hydroxyethyl methyl cellulose with viscosity of 25,000 mPa·s and 45,000 mPa·s (for measurements using a Brookfield rheometer at 20 °C) with low chemical modification. This admixture has the form of white powder with a grain size below 0.063 mm. The pH of its 1% solution in water (at 20 °C) is 7.0. The characteristics of cement and hydrated lime are given in Table 1 and in Table 2, respectively.

### 2.2. Preparation of Mortars and Pastes

Mortars and pastes differing in their composition with the type of binder, amount, and viscosity of HEMC admixture were prepared for the tests. As a reference material, a cement mortar without admixture, designated as C-0, was used. In addition, cement composites modified with admixture in various amounts and viscosity (with symbols C-0.05-MV, C-0.3-MV, and C-0.175-HV) and composites modified with cellulose ether, in which cement was replaced with 50% hydrated lime (marked with symbols CL-0.05-MV, CL-0.3-MV, and CL-0.175-HV). The MV symbol stands for Medium Viscosity (cellulose ether with a viscosity of 25,000 mPa·s), whereas the HV symbol stands for High Viscosity (cellulose ether with viscosity of 45,000 mPa·s). The admixture was added in the amount of 0.05%, 0.175%, and 0.3% in the relation to the dry ingredients of the mortar (binder and fine aggregate). The detailed composition of mortars is given in Table 3. The article is a part of a wider study concerning the analyzed issue in which the influence of cellulose ethers and hydrated lime on selected properties of plastering mortars was studied [31]. Some research in this direction has been published in articles [14,15,30]. The composition of pastes was similar to the composition of mortars, excluding fine aggregate.

Cement, hydrated lime, and fine aggregate were weighed with an accuracy of 0.1 g, and the chemical admixture was weighed with an accuracy of 0.0001 g. The ingredient mixing procedure was as follows: 90 seconds of mechanical mixing at 45 rpm, 30 s of rest, and 90 s of mechanical mixing at 57 rpm. The time and method of mixing were constant (the same in each case).

All mortars were performed, maintaining a constant weight ratio of the binder to the fine aggregate of 1:10. The amount of water was chosen so as to obtain a constant consistency of 165 mm, measured using a flow table, according to the PN-EN 1015-3:2000 standard [32]. All samples were prepared and tested in an air-conditioned laboratory at a temperature of 20 ± 2 °C and a relative air humidity of 65 ± 5%.

### 2.3. Methods

Determination of the effect of hydroxyethyl methyl cellulose on the setting processes of cement and cement-lime composites was carried out using three methods, which included:

- Testing of setting processes by the ultrasonic method,
- Calorimetric measurements,
- Phases analysis by the XRD method.

Using the first method, mortar samples were tested, whereas, using the second method, paste samples were tested. In each case, the experiments were carried out for the first 48 h of material maturation. Additionally, using X-ray diffraction analysis, measurements after 40 days of maturation were made to verify whether there are differences in the leaven hydration products compared to the results obtained after two days.

**Figure 1:**
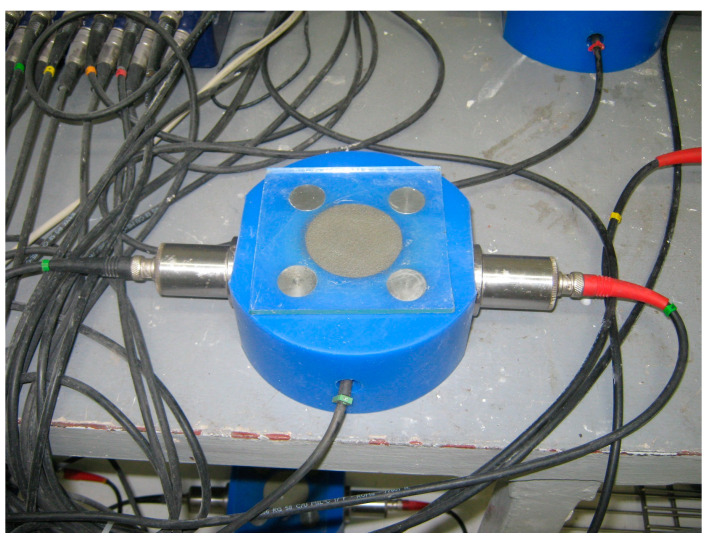
Mortar sample during ultrasonic testing.

The setting process was determined using the ultrasonic wave velocity method by an IP-8 Ultrasonic Measuring System (UltraTest GmbH, Achim, Germany). The essence of the study consisted in passing the ultrasonic wave through the material from one end to the other and recording its velocity over time. Ultrasonic heads and a temperature sensor were mounted on both sides for all forms. Each time, a sample of fresh mortar was taken (a cylindrical sample with a height of 5 cm and a diameter of 5 cm), which was placed for the entire duration of the test in the sampler (Figure 1). The upper surface of the tested material was protected with a glass plate to limit its water loss through drying. The change in the velocity of the ultrasonic wave and the temperature inside the samples were recorded every 60 s for a period of 48 h of measurements and saved automatically in the computer’s memory.

The BT2.15CS low-temperature differential scanning microcalorimeter by SETARAM (SETARAM, Plan-les-Ouates, Geneva, Switzerland) was used for micro-calorimetric measurements. Binder samples (with admixture or without it) were mixed with distilled water and put into small PE zip-bags and, immediately after mixing, placed in the calorimeter. Calorimetric curves were recorded with an electronic measuring unit, connected to a personal computer.

XRD images were obtained in an X-ray diffractometer Empyrean (PANalytical, Almelo, Netherlands), equipped with a copper lamp. FT scanning was carried out at a 2θ range of 5–65° and the step length was 0.0167°. The PANalytical XRD analysis software HighScore 4.6 (PANalytical, Almelo, Netherlands) with the International Center for Difration Data (ICDD) database PDF-2 was used for phase identification. X-ray diffractometric studies were carried out on powdered samples of pastes after the time of 2 and 40 days of setting.

## 3. Results and Discussion

### 3.1. Effect of Setting Processes by the Ultrasonic Method of Mortars

**Figure 2:**
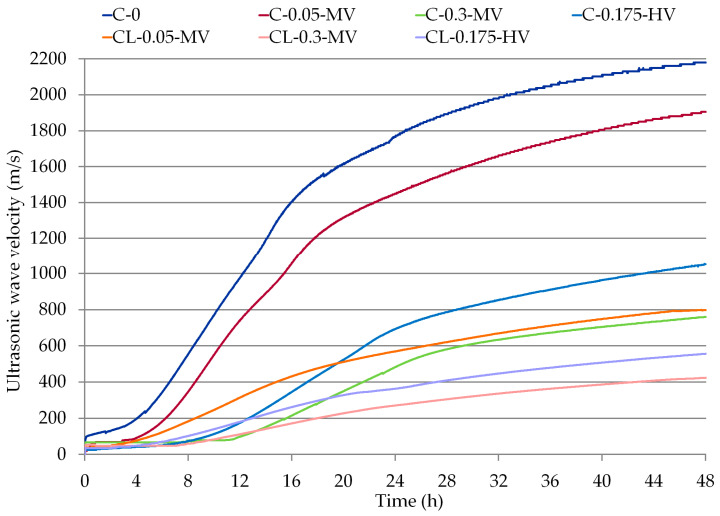
Ultrasonic wave velocity changes in all mortars (according to Table 3).

The ultrasonic technique used in the research is based on the registration of the speed of the ultrasonic wave, which is related to the change in the viscosity and elasticity of the medium. The publications [14,30,33,34,35,36] show the possibilities of using this method. Since the author rightly confirmed [33], the ultrasonic technique is used to evaluate the quality of ready products. A relatively new area of research where this method can be used is the examination of the setting and hardening processes of cement-based materials. According to Zych [33], due to changes in visco-elastic properties caused by hardening, the velocity of wave propagation starts growing. Applying this method, it is possible to describe the rate of setting and hardening of mortars based on cement and cement-lime binders and it is possible to assess the influence of the admixture on the setting processes, which might be useful for assessing the processing time of plastering mortars when choosing a method or establishing the conditions of building, repair, and assembly works [14,33]. The results of measurements obtained with the use of the ultrasonic method, presented in Figure 2 and in Table 4, prove the different properties of mortars. There is a clear influence of the admixture on the reduction of the ultrasonic wave velocity during the entire tested measurement. Thus, it can be concluded that cellulose ether affects the mortar setting processes. In the case of cement mortars, Kulesza et al. [36] obtained similar results. However, the authors focused their research on evaluating the influence of re-dispersible powders on setting and hardening processes of thin-bed mortars. The influence of cellulose ether was assessed by them as a secondary effect. As for the monitoring of the setting processes of cement-lime mortars, no reference articles were found in the literature.

The setting process varies in intensity depending on the binder used and the amount and viscosity of the cellulose ether. It is clearly visible that the use of an admixture in the amount of 0.3% delays the setting process of both cement and cement-lime mortars (C-0.3-MV and CL-0.3-MV mortars). This is especially true when comparing the induction period of individual materials. This parameter is defined as the time counted from the setting of the measurement to the moment of a sudden change in the velocity of the ultrasonic wave. It should also be noted that, in the case of all modified mortars, the maximum velocity of the ultrasonic wave has been significantly reduced, from 2180.1 m/s, even to 423.3 m/s (more than 5 times), which may be related to the different microstructure (among other porosities) of these materials, resulting from the replacement of cement binder on lime and the use of admixtures. Mortar CL-0.3-MV modified with cellulose ether, in which 50% cement was replaced with hydrated lime, was characterized not only by the lowest ultrasonic wave velocity after 48 h of maturation, but also the ultrasonic wave velocity recorded during all setting processes was the lowest. The smallest differences in the setting process, in relation to the reference sample (C-0), are visible for the cement mortar with the admixture of 0.05% (sample C-0.05-MV) and viscosity of 25,000 mPa·s. In other cases, it is clearly visible that both the lime and the admixture significantly reduce the velocity of the ultrasonic wave during every period of time for measurements. The induction period of 10 h 54 min and 7 h 18 min indicates that the setting processes of C-0.3-MV and CL-0.3-MV mortars were delayed due to the use of an admixture in the amount of 0.3% and an admixture viscosity of 25,000 mPa·s. The induction time is visible only in the case of mortars modified with a cellulose ether of 0.175% and 0.3%. Despite the fact that the induction time is clearly visible only in the case of four mortars (Table 4), a slow increase in the ultrasonic velocity was recorded in the initial periods for the remaining materials. It is clearly visible that the most important factor in delaying the setting process is the use of admixtures, which is not so noticeable, even in the case of replacing a part of the cement binder with hydrated lime. When testing with the ultrasonic method, the amount of the admixture had a greater influence on the setting process than its viscosity. When analyzing this process for C-0.3-MV and CL-0.05-MV mortar, it is worth noting that, although the ultrasonic wave velocity recorded after 48 h is at a similar level, the induction period and the setting course in the first 12 h are different, which only confirms the conclusions regarding the influence of the amount of the admixture on the processes taking place during the maturation of these materials.

### 3.2. Effect of Hydration by the Calorimetry Method of Pastes

**Figure 3:**
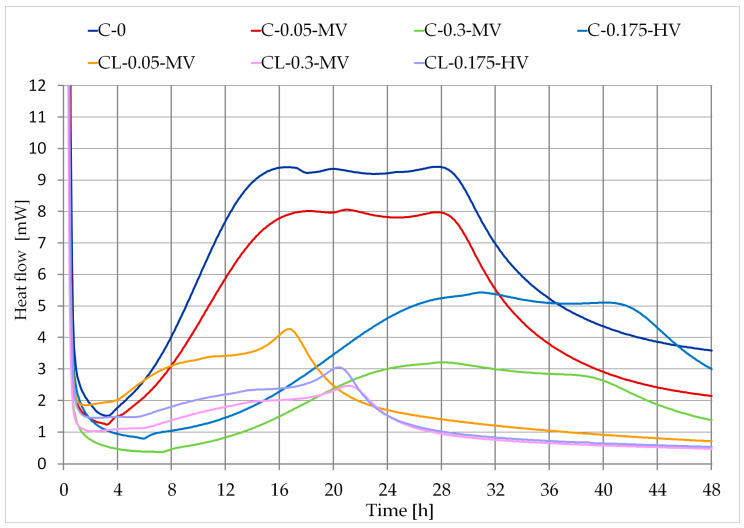
Isothermal calorimetry curves of all pastes (according to Table 3).

**Figure 4:**
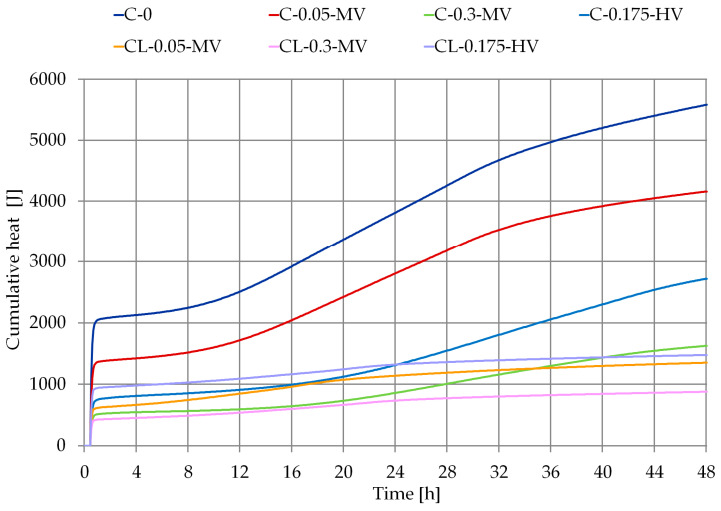
Cumulative curves of all pastes (according to Table 3).

The changes in the rate of heat release and the amount of heat released during the hydration of pastes as a function of time are shown in Figure 3 and Figure 4. In the case of C-0 paste, there is a clear peak in the heat flow diagram (Figure 3). In the first 16 h, a rapid hydration reaction was noted, which was confirmed by using the ultrasonic method. In Figure 2, the setting course of the C-0 sample was characterized by a rapid increase in the ultrasonic wave velocity. Based on the micro-calorimetric measurements, it can be concluded that the admixture delays the hydration processes and extends the induction period, which was also observed in literature [10,22,24,37]. The influence of the admixture on the delay of the hydration process is related to the specificity of this admixture–its water retention capacity [2,3,6,10]. Adding a cellulose ether changes the viscosity of the pastes [5,6,9,16] as well as the processes of cement hydration [19,23,24]. The amount of cellulose ether is essential when changing heat flow. Replacing a part of the cement binder with hydrated lime also reduces the heat release energy throughout the measurement and the induction period is also extended. When analyzing Figure 3, it can be noticed that pastes C-0 and C-0.05-MV have three distinct peaks in the curve, pastes C-0.3-MV and C-0.175-HV–two peaks, but pastes CL-0.05-MV, CL-0.3-MV, and CL-0.175-HV have only one clear peak. One peak on curves for this pastes may be caused by replacing part of the cement binder with hydrated lime.

The curve of cumulative heat of hydration (Figure 4) shows that cellulose ether reduces the maximum rate of heat release in 48 h, which was observed in the research [10,24,25]. Cumulative heat after 48 h was reduced by up to 76% in the case of cement pastes and up to 79% in the case of cement-lime pastes (Table 5). The polymer admixture extended the induction time from 2h 50 min to 13 h (in the case of cement pastes) and from 2 h 50 min to 5 h 15 min (in the case of cement-lime pastes). Adsorption between cellulose ethers and hydration products is thought of as the major cause for the retardation, which acts between OH groups of cellulose ethers and metal hydroxides on the surface of the hydration products [25].

In Table 5, some parameters relating to the heat evolution curves were noted. Induction time was read based on Figure 3, but the time of maximum and cumulative heat were read based on Figure 4.

Comparing the results obtained on the basis of measurements using the ultrasonic technique with the results of calorimetric tests, there can be a similarity of the course of the curves showing the change in the velocity of the ultrasonic wave in time (Figure 2, Table 4) and the course of the curves of cumulative heat of hydration (Figure 4, Table 5). Mortar C-0.3-MV and paste C-0.3-MV have the longest induction time, which was measured by an ultrasonic technique and calorimetric measurements. The differences visible in the diagrams of changes in the velocity of the ultrasonic wave of individual mortars (Figure 2) are similar to the diagram of accumulated heat during the hydration processes of pastes (Figure 4).

### 3.3. Effect of Hydration by the X-ray Diffraction of Pastes

**Figure 5:**
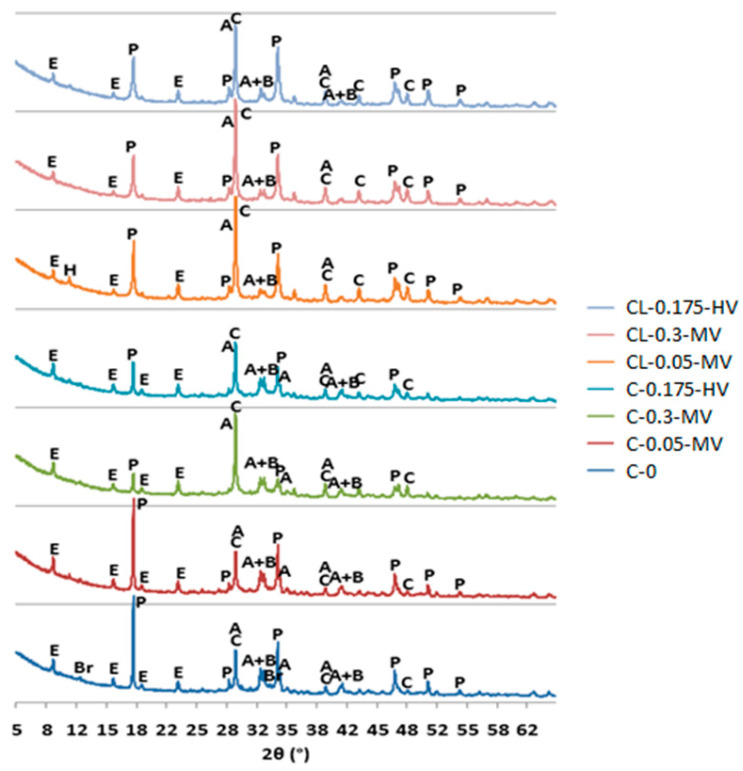
X-ray patterns of all pastes for 48 h. Denotation: A–alite, B–belite, Br–brownmillerit, C–calcite, E–ettringite, P–portlandite, H–C-S-H.

The hydration of the reference paste (cement paste without admixture of cellulose ether) is model-like, which means that, in this case, significant amounts of Portlandite are formed (Figure 5). The largest amount of Portlandite in the C-0 paste and the smallest intensity of the peaks of the phases of alite and belite are related to their faster reaction as a result of the hydration process, which was confirmed in calorimetric studies [38,39]. Diffraction patterns of pastes modified with cellulose ether after 48 h of setting indicate that the admixture inhibits the hydration process of cement and cement-lime pastes. In the case of the diffractograms of the samples from C-0.3-MV to CL-0.175-HV, a lower intensity of Portlandite can be seen compared to the diffractogram of the C-0 and C-0.05-MV pastes. It can be seen that the hydration of the cement paste with an admixture of 0.05% with a viscosity of 25,000 MPa·s (C-0.05-MV) does not differ from the hydration of the C-0 paste. Hence, the conclusion that HEMC added in the amount of 0.05% does not have such a large impact on the setting process as in other cases. The lowest amount of Portlandite was observed for the cement paste modified with an admixture of 0.3% with a viscosity of 25,000 MPa·s. These experiments confirmed that the admixture viscosity does not have such a large influence on the changes in the hydration process as its amount, regardless of the type of binder.

For cement-lime pastes, the increase of calcite peaks is also characteristic, which may indicate of the partial lime binder carbonation. Due to a binder composition, alite and belite have less intense peaks in these pastes.

**Figure 6:**
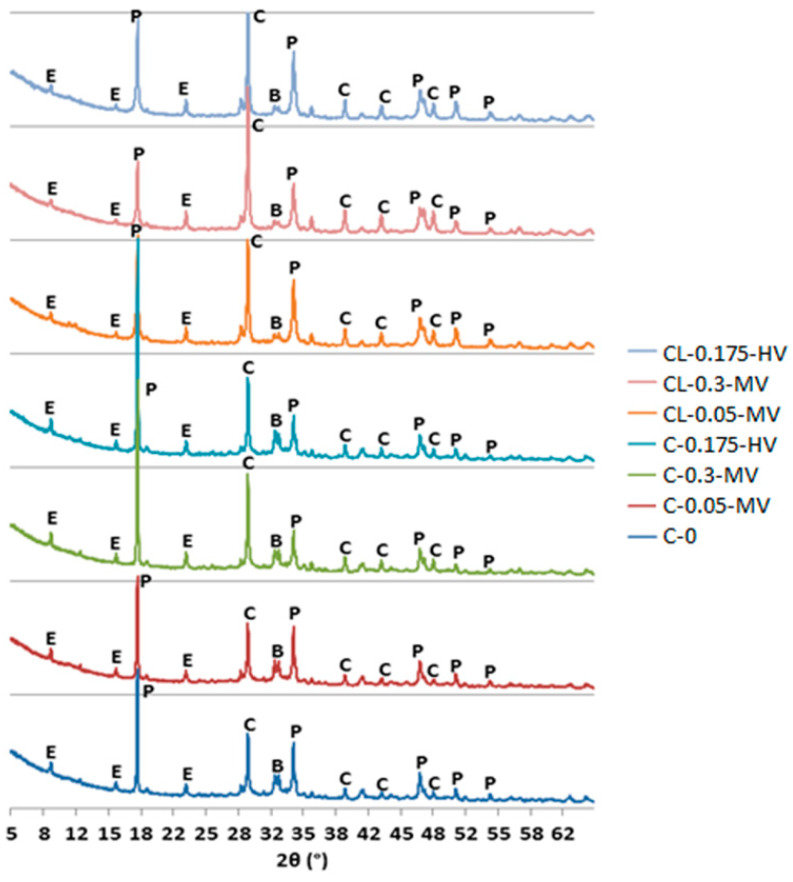
X-ray patterns of all pastes for 40 days. Denotation: A–alite, B–belite, Br–brownmillerit, C–calcite, E–ettringite, P–portlandite.

The diffractograms of the pastes after 40 days of maturation were shown in Figure 6. It can be seen that the process of setting individual pastes is similar. There are few significant differences (between the individual pastes) in the intensity of portlandite, alite, and belite peaks, as could be observed for the measurements carried out after two days of hydration of the samples. A similar conclusion for cement pastes was observed in literature reports [22]. Only for the C-0.3-MV and C-0.175-HV pastes, differences for the characteristic peaks of Portlandite are clearly visible. In both cases, the peak for 18.04° 2θ is significantly higher and the peak for 34.05° 2θ is lower than in the other pastes. The least intense Portlandite peak was obtained for CL-0.3-MV.

However, comparing the diffraction patterns after two days and after 40 days, it is possible to notice the different intensity of Portlandite and calcite. A greater amount of Portlandite after 40 days may be caused by the hydration process, and an increased amount of calcite is associated with the formation of calcium carbonate CaCO_3_ and the progressive carbonation process. In Table 6, the percentage changes of the intensity of Portlandite peaks for 18.04° 2θ after 40 days of pastes hydration were shown. In the case of calcite, the difference in peak intensity between the diffractograms made after two days and after 40 days is also visible, although not as large as in the case of Portlandite. However, the results are consistent with the studies carried out by Izaguirre et al. [27]. In the case of the sample with cellulose ether, a significant increase in the amount of free water, Ca(OH)_2_ and CaCO_3_ were seen in TG analysis. Percent content H_2_0, Ca(OH)_2_, and CaCO_3_ after 28 and 91 days was bigger than after 7 days maturation (compared to a reference sample), which confirm the ongoing carbonation processes. On the other hand, percent content of Ca(OH)_2_ and CaCO_3_ after 7 days was smaller in the case of the sample with cellulose ether than the reference sample, which may indicate a delay of reaction of carbonation processes caused by the admixture.

## 4. Conclusions

The following conclusions could be drawn from the research.

- The addition of hydroxyethyl methyl cellulose influences the setting processes of cement and cement-lime mortars. The addition of HEMC with a viscosity of 25,000 mPa·s in the amount of 0.3% significantly delays the setting process regardless of the type of binder (cement, hydrated lime), which is not so visible in the case of the admixture in the amount of 0.05%.
- HEMC reduces the rate of the hydration reaction in cement and cement-lime pastes, which has been confirmed in calorimetric tests. The induction time on the heat evolution curve becomes significantly longer (by a maximum of 10 h 10 min). The changes are the most visible with an increasing dosage of the admixture.
- In the XRD analysis, significant effects of the presence of HEMC were found on the resulting Portlandite peaks. Limiting their intensity during the period of the first two days shows that the hydration process is lower.
- The differences in the hydration of pastes after 40 days of maturation are much smaller than the hydration of the same samples after two days of maturation. This shows that significant phase changes take place at a later stage of hydration (after 48 h). The addition of HEMC has a greater effect on the hydration of cement and cement-lime pastes in the initial hours of maturation. After a longer time, this effect practically disappears. Therefore, control of hydration kinetics of cement and cement-lime pastes on early stages is important.
- The effect of HEMC admixture on the inhibition of setting processes is more visible in pastes and mortars containing only cement binder, especially by analyzing the differences in the induction time from the ultrasonic technique and calorimetric measurements.
- The amount of the admixture used is the most important in the process of setting pastes and mortars. A much-reduced impact is visible when taking into account the viscosity of the admixture or the type of binder used.
- The ultrasonic technique in combination with calorimetric measurements and XRD method gives the possibility of a more complete and broader assessment of the setting mortars. It also enables the assessment of the influence of a chemical admixture on the hydration and setting processes of pastes and mortars. It can be useful in monitoring and controlling for application plastering mortars.

## Figures and Tables

**Table 1:** Chemical composition and physical properties of cement.

Chemical Composition (wt. %)		Physical Properties	
SiO_2_	20.22	Water requirement of normal consistency/%	28.8
Al_2_O_3_	4.43	Initial setting/min	173
Fe_2_O_3_	3.65	Final setting/min	237
CaO	64.06	Specific surface area/(m^2^/kg)	387.9
Na_2_O	0.29	2 d compressive strength/MPa	28.9
MgO	1.24	28 d compressive strength/MPa	59.5
SO_3_	3.31	2 d flexural strength/MPa	5.1
Cl	0.093	28 d flexural strength/MPa	8.4
K_2_O	0.54	Loss on ignition/%	3.81
free CaO	1.83	-	-

**Table 2:** Chemical composition of hydrated lime.

Chemical Composition (wt. %)	
CaO + MgO	95.17
MgO	0.80
CO_2_	1.86
SO_3_	0.41

**Table 3:** Composition of mortars.

Mortar/Paste	Binder (Cement + Lime) (g)	Fine Aggregate (g)		Cellulose Ether (wt.%)	Viscosity of Cellulose Ether	Water (g)
		0.1–0.5 mm	0.2–0.8 mm		(mPa·s)	
C-0	96	437	467	0.00	-	195
C-0.05-MV	96	437	467	0.05	25,000	148
C-0.3-MV	96	437	467	0.30	25,000	220
C-0.175-HV	96	437	467	0.175	45,000	185
CL-0.05-MV	48 + 48	437	467	0.05	25,000	176
CL-0.3-MV	48 + 48	437	467	0.30	25,000	225
CL-0.175-HV	48 + 48	437	467	0.175	45,000	195

**Table 4:** Comparison of results read from the graphs (Figure 2).

Mortar	Induction Time (h)	Ultrasonic Wave Velocity			
		after 12 h (m/s)	after 24 h (m/s)	after 36 h (m/s)	after 48 h (m/s)
C-0	0 h 00	980.6	1765.7	2047.5	2180.1
C-0.05-MV	0 h 00	822.1	1478.7	1740.4	1905.3
C-0.3-MV	10 h 54	98.9	484.0	673.2	760.0
C-0.175-HV	3 h 40	174.9	693.3	911.9	1050.4
CL-0.05-MV	0 h 00	315.6	570.0	712.3	797.3
CL-0.3-MV	7 h 18	112.0	270.4	363.2	423.3
CL-0.175-HV	2 h 04	181.0	363.2	479.3	556.4

**Table 5:** Characteristic parameters of heat evolution curves for all pastes (Figure 3 and Figure 4).

Pastes	Induction Time (h)	Time of Second Maximum (h)	Cumulative Heat			
			after 12 h (J)	after 24 h (J)	after 36 h (J)	after 48 h (J)
C-0	2 h 50	16 h 35	2555.49	3870.52	5004.47	5583.49
C-0.05-MV	3 h 10	18 h 15	1753.67	2855.17	3781.15	4160.72
C-0.3-MV	13 h 00	28 h 10	598.18	878.04	1316.79	1625.28
C-0.175-HV	4 h 45	31 h 00	919.00	1341.37	2088.41	2718.38
CL-0.05-MV	4 h 30	16 h 40	861.95	1146.33	1271.30	1351.04
CL-0.3-MV	5 h 15	21 h 05	545.89	740.88	826.38	878.06
CL-0.175-HV	4 h 50	20 h 20	1100.67	1327.26	1418.96	1476.46

**Table 6:** Percentage changes in Portlandite intensity after 2 and 40 days of maturation for all pastes (according to Table 3).

Pastes	Peak Intensity after 2 Days (%)	Peak Intensity after 40 Days	Changes in Peak Intensity
		(%)	(%)
C-0	100.00	100.00	+15.60
C-0.05-MV	98.58	83.43	−2.17
C-0.3-MV	19.44	149.50	+788.85
C-0.175-HV	33.77	172.03	+488.81
CL-0.05-MV	58.96	83.26	+63.25
CL-0.3-MV	44.87	50.65	+30.48
CL-0.175-HV	44.58	73.40	+90.33
